# Correlation of histological and microbiological findings in septic and aseptic knee implant failure

**DOI:** 10.1007/s00402-019-03159-x

**Published:** 2019-03-11

**Authors:** Y. Inagaki, Y. Uchihara, M. Munemoto, M. Scarborough, C. A. F. Dodd, C. L. M. H. Gibbons, Y. Tanaka, N. A. Athanasou

**Affiliations:** 1 Nuffield Department of Orthopaedics, Rheumatology and Musculoskeletal and Clinical Laboratory Services, University of Oxford, Nuffield Orthopaedic Centre, OX3 7HE Oxford, UK; 2 Department of Orthopaedic Surgery, Nara Medical University, Shijocho 840, 634-8521 Kashihara, Japan

**Keywords:** Knee, Arthroplasty, Infection, Histology, Microbiology, MSIS

## Abstract

**Introduction:**

The Musculoskeletal Infection Society (MSIS) has defined specific clinical and laboratory criteria for the diagnosis of periprosthetic joint infection (PJI). In this study we assessed the diagnostic utility of MSIS microbiological and histological criteria for PJI in 138 cases of septic and aseptic knee implant failure.

**Materials and methods:**

Intra-operative samples from 60 cases of knee septic implant failure (SIF) and 78 cases of aseptic implant failure (AIF), defined on the basis of clinical, laboratory and operative findings/surgical management, were analysed microbiologically and histologically. Findings were correlated with the final clinical diagnosis and the specificity, sensitivity, accuracy, positive and negative predictive value of MSIS microbiological and histological criteria for knee PJI were assessed.

**Results:**

80% of SIF cases showed culture of the same organism from two or more samples (ie MSIS microbiological criteria for definite PJI); 8.3% grew an organism from one sample, and 11.7% showed no growth from any sample. 23.1% of AIF cases grew an organism from one sample and 76.9% showed no growth from any sample. MSIS histological criteria for PJI identified 96.7% of SIF cases. The sensitivity, specificity, accuracy and positive and negative predictive value of MSIS histological criteria for PJI were 96.7%, 100%, 98.6%, 100% and 97.5%, respectively. MSIS microbiological and histological criteria identified all AIF cases.

**Conclusions:**

Knee PJI is more often identified by current MSIS histological than microbiological criteria. A significant proportion of SIF cases show either no growth or growth of an organism from only one sample. AIF is identified by both MSIS microbiological and histological criteria. Correlation of clinical, radiological and laboratory findings is required for the diagnosis of knee PJI.

## Introduction

Periprosthetic joint infection (PJI) is a significant cause of failure of primary knee arthroplasty [1]. Clinical findings and the results of laboratory tests, radiological investigations and microbiological/histological examination of synovium and synovial fluid are used to diagnose knee PJI pre-operatively [2, 3], whilst intra-operative findings and post-operative microbiology and histology results are used to confirm the diagnosis. Accurate diagnosis of septic implant failure (SIF) and aseptic implant failure (AIF) is required to ensure correct surgical / medical management. Misdiagnosis of SIF (or AIF) has significant clinical and cost implications in terms of implant survival, patient morbidity, the need for prolonged antibiotic therapy, length of hospital stay and consideration of the need for a second surgical procedure [4–6].

The Musculoskeletal Infection Society (MSIS) has defined criteria for the diagnosis of PJI on the basis of clinical, microbiological, histological and other laboratory findings [7, 8], MSIS criteria for a definite diagnosis of PJI are either the presence of a sinus tract or the microbiological culture of a phenotypically indistinguishable organism from two or more separate tissue or fluid samples obtained from a prosthetic joint. In addition to these two ‘major’ criteria, the former MSIS criteria specify five ‘minor’ criteria for the diagnosis of PJI: when three of these criteria are met, then it is considered that there is sufficient evidence for a definite diagnosis of PJI. Microbiological culture of an organism from one sample of periprosthetic tissue or fluid and the histological finding in periprosthetic tissue of more than 5 neutrophil polymorphs (NPs) per (×400 magnification) high power field (HPF) are two of these minor criteria. A recent evidence-based analysis of MSIS and other diagnostic criteria found that positive histology (ie > 5 NPs per HPF) strongly correlated with the diagnosis of PJI [9]. It has, however, been noted that SIF periprosthetic tissues can contain fewer NPs in some cases [10–13].

In a previous study on hip implant failure (which pre-dated the formulation of MSIS criteria for the diagnosis of PJI), we found that histology correlated more closely than microbiology with regard to the final clinical diagnosis of SIF [10]. Although the value of histology in the assessment of arthroplasties has been shown in recent studies [3, 10–14], there is controversy regarding its relative importance in establishing this diagnosis of PJI, and, with regard to knee arthroplasties, histological parameters for this diagnosis have not formally been defined. In this study, we have compared histological and microbiological findings in 138 cases of knee arthroplasty failure diagnosed and treated as SIF or AIF. Our aim has been not only to determine whether histology or microbiology findings correlate more closely with the final clinical diagnosis of SIF or AIF but also to reassess the utility of specific MSIS microbiology and histology criteria for the diagnosis of knee PJI.

## Patients and methods

### Knee SIF and AIF cases sampled

The case notes and results of pre-operative laboratory investigations and intra-operative findings of 138 revision knee arthroplasty cases were reviewed. These included 116 total knee replacements, 19 unicompartmental knee replacements derived from 73 females and 65 males, with mean age 69.1 years (range 36–95 years). In all cases, which were consented for research, the original knee arthroplasty was carried out for osteoarthritis. The cases were classified after review at a multidisciplinary meeting into those in which the final diagnosis (on which treatment was based) was SIF or AIF. In addition to noting pre-operative clinical and radiological findings suggestive of PJI, SIF cases fulfilled at least two of the following criteria: (1) raised CRP level, (2) positive pre-operative joint aspiration culture, (3) positive pre-operative joint histology, (4) the presence of a sinus and (5) purulent intraoperative tissue. SIF cases had a two-stage revision arthroplasty and were treated with long-term antibiotics. AIF cases (which did not fulfil two of the above criteria) had a one-stage revision arthroplasty and recovered fully without the need for antibiotics.

In all the above cases, discrete (non-identical) samples of periprosthetic tissue (knee joint capsule, femoral and tibial pseudomembrane) were submitted for microbiology and histology and taken at the time of revision arthroplasty [15].

### Microbiology analysis

At least four independent specimens of knee joint periprosthetic tissue from the joint capsule and arthroplasty membrane were submitted and cultured by direct and enriched methods as previously described [15]. Briefly, specimens were transferred to universal receptacles containing 3 ml of peptone broth and Ballotini glass balls. The tissue was disrupted by shaking. Aliquots of the processed tissue in peptone were plated onto two blood agar plates (5% horse blood)—one aerobic and one anaerobic and onto a chocolate agar plate. The remainder of the tissue was transferred to tryptone soy broth, 0.1% agar for enrichment culture. Aerobic and anaerobic cultures were incubated for 14 days and organisms and antibiotic sensitivities identified as previously described [15]. Isolates were considered significant if an indistinguishable strain grew in more than one independent sample culture. Microbiological findings were grouped into those where:

1. Cultures showed growth of a phenotypically indistinguishable organism from two or more independent samples;
2. Cultures showed growth of an organism from only one sample, the other samples showing no significant growth;
3. Cultures showed no growth in any of the samples.

### Histology analysis

Samples of knee joint periprosthetic tissue were fixed in 10% neutral buffered formalin, routinely processed with 5 µm paraffin sections cut and stained with haematoxylin and eosin. Chloroacetate esterase (CAE) staining was carried out as previously described [16] in all cases diagnosed clinically as PJI and in all cases where NPs were identified histologically. The sections were examined to find areas of maximum inflammatory cell infiltration and the extent of the NP infiltrate in these areas scored semi-quantitatively as previously described [10, 11]. At least five HPF (×400) in five different areas of each histological section (ie 25 HPF) were examined and the number of NPs in these areas counted. From this, the average number of NPs per HPF was calculated. NP infiltration was scored as follows: 0 = no polymorphs identified; 1+ = < 1 NPs per HPF; 2+ = 1–5 NPs per HPF; 3+ = > 5 NPs per HPF.

### Data analysis

To determine the correlation of histological and microbiological findings with the final clinical diagnosis, the specificity, sensitivity, accuracy, positive predictive value (PPV) and negative predictive value (NPV) of the microbiological and histological findings for SIF was determined as previously described [10, 11]. Sensitivity was calculated as the proportion of cases with a final clinical diagnosis of SIF correctly identified by specific MSIS microbiology or histology criteria, i.e. the number of true positive results divided by the sum of true positive and false negative results. Specificity was calculated as the proportion of cases with a final diagnosis of AIF correctly identified by specific microbiology or histology criteria, i.e. the number of true negative results divided by the sum of true negative and false positive results. Accuracy was calculated as the ratio of true positive and true negative results to the total number of results. The PPV was calculated as the ratio of true positive results to the total number confirmed as infected by histology or microbiology criteria. The NPV was calculated as the ratio of true negative results to the number of cases found not to be infected by histology or microbiology criteria.

## Results

Of the 138 knee arthroplasties examined in this study, 60 cases had a final clinical diagnosis of SIF and 78 cases of AIF. Correlation of the final clinical diagnosis with the findings on microbiology and histology from intra-operative samples is shown in Table 1. The number and range of organisms isolated from two or more samples in cases of definite PJI are shown in Table 2.

**Table 1:** Knee SIF and AIF: correlation with microbiology and histology

Final diagnosis	Microbiology	Histology
Knee SIF 60 cases (100%)	No growth: 7 cases (11.7%) One positive sample^a^: 5 cases (8.3%) Two or more positive samples^b^ : 48 cases (80.0%)	0: 1 case (1.7%) 1+: 0 cases (0%) 2+: 1 case (1.7%) 3 + ^c^: 58 cases (96.7%)
Knee AIF 78 cases (100%)	No growth 60 cases (76.9%) One positive sample^a^ 18 cases (23.1%) Two or more positive samples^b^ 0 cases (0%)	0: 66 cases (84.6%) 1+ : 10 cases (12.8%) 2+ : 2 cases (2.6%) 3 + ^c^: 0 cases (0%)

^a^MSIS (minor) microbiological criteria supportive of the diagnosis of PJI

^b^MSIS (major) microbiological criteria for the definite diagnosis of PJI

^c^MSIS histological criteria supportive of the diagnosis of PJI

**Table 2:** Micro-organisms isolated from two or more samples in knee SIF cases

Staphylococcus aureus (12)
Staphylococcus epidermidis (12)
Sterptococcus species (8)
Enterococcus species (6)
Coagulase negative staphylococcus (4)
*Escherichia coli* (3)
Proteus species (3)
Staphylococcus species (3)
Morganella species (2)
Candida species (2)
Citrobacter species (2)
Other^a^ (6)

In five SIF cases more than one species of micro-organisms was isolated

^a^*Bacillus pumilus* (1), *Bacteroides fragilis* (1), *Dermabacter hominis* (1), *Enterobacter cloacae* (1), *Finegoldia magna* (1), *Propionibacterium species* (1)

( ) = number of SIF cases

### Correlation of the final clinical diagnosis of knee SIF and AIF with microbiological and histological findings

Major and/or minor MSIS microbiological criteria for PJI were met in most cases where the final diagnosis was SIF, although in 11.7% of SIF cases there was no growth of organisms. Histological identification or 3+ NPs (i.e, more than five NPs per HPF) was noted in almost all SIF cases. In all 78 cases that had a final clinical diagnosis of AIF, major MSIS microbiological criteria for PJI were not met. Most of these cases showed no growth from any sample but 23.1% showed growth of an organism from one sample. Histological analysis showed that NPs were absent in most AIF cases with none showing 3+ NPs. The sensitivity, specificity, accuracy, NPV and PPV of MSIS (major and minor) microbiological and histological diagnostic criteria for the final clinical diagnosis of SIF and AIF are shown in Table 3. It was noted that MSIS histological criteria of 3+ NPs were of considerable diagnostic utility in identifying SIF/AIF, showing higher sensitivity, accuracy and NPV than major microbiological criteria.

**Table 3:** Data analysis: MSIS microbiological and histological criteria

	Microbiological culture of pathogen from one sample^a^ (%)	Microbiological culture of pathogen from two or more samples^b^ (%)	Histological identification of 3+ NPs^c^ (%)
Sensitivity	88.3	80.0	96.7
Specificity	76.9	100	100
Accuracy	81.9	91.3	98.6
PPV	74.7	100	100
NPV	89.6	86.7	97.5

^a^MSIS (minor) microbiological criteria supportive of the diagnosis of PJI

^b^MSIS (major) microbiological criteria for the definite diagnosis of PJI

^c^MSIS histological criteria supportive of the diagnosis of PJI

### Correlation of microbiological and histological findings

In almost all of the cases (97.9%) identified as definite PJI by major MSIS microbiology criteria, histology showed the presence of more than 5 NPs per HPF on average (Table 4). The single case in which this was not noted contained three NPs per HPF on average in periprosthetic tissue. No organism was cultured from any sample microbiologically in 67 cases, 60 of which showed fewer than 5 NPs per HPF histologically. In most of these cases, the NP infiltrate scored 0 (51 cases) or 1+ (8 cases). However, there were seven cases in this group, all of which were clinically diagnosed as SIF, which showed 3+ NPs on histology. In 23 cases where an organism was cultured microbiologically from only one sample, histology showed 3+ NPs in four cases and 2+ NPs in one case. These six cases were clinically diagnosed as SIF. In the remaining 18 cases, NPs were absent or 1+ NPs were noted; all these cases had a final clinical diagnosis of AIF. In all cases where one sample on microbiology culture was positive and, histology showed more than 2+ NPs, the final clinical diagnosis was SIF.

**Table 4:** Microbiology findings: correlation with histology and final diagnosis

Microbiology	Histology	Final diagnosis
No growth—67 cases (100%)	0: 51 cases (76.1%) 1+: 8 cases (11.9%) 2+: 1 case (1.5%) 3+ ^c^: 7 cases (10.4%)	SIF: 7 cases (10.4%) AIF: 60 cases (90.0%)
One sample positive^a^—23 cases (100%)	0: 16 cases (70.0%) 1+: 2 cases (8.7%) 2+: 1 case (4.3%) 3+ ^c^: 4 cases (17.4%)	SIF: 5 cases—(21.7%) AIF: 18 cases (78.3%)
Two or more positive samples^b^—48 cases (100%)	0: 0 cases (0%) 1+: 0 cases (0%) 2+: 1 case (2.1%) 3+ ^c^: 47 cases (97.9%)	SIF: 48 cases—(100%) AIF: 0 cases (0%)

^a^MSIS (minor) microbiological criteria supportive of the diagnosis of PJI

^b^MSIS (major) microbiological criteria for the definite diagnosis of PJI

^c^MSIS histological criteria supportive of the diagnosis of PJI

## Discussion

Microbiology and histology investigations on tissue samples taken at the time of revision knee surgery provide diagnostic information on the cause of knee implant failure, particularly in distinguishing between SIF and AIF. There have been relatively few studies which have focused on the role of histology in knee implant failure. Most previous studies have evaluated histological findings in combined series of hip and knee arthroplasties with a view to determining whether intra-operative frozen section histology is useful in the diagnosis of PJI with no breakdown of the results for hip and knee arthroplasties. Kwiecien et al. [17] studied the frozen section histology of 100 knee and 100 hip arthroplasties and found high specificity, PPV, NPV, accuracy and moderate sensitivity for the diagnosis of PJI; this study also noted 97.6% concordance between the histology results on frozen sections and fixed paraffin embedded sections. Borrego et al. [18] similarly studied 83 hips and 63 knees and found good correlation of the NP count with the microbiological results of PJI but noted that the absence of NPs did not exclude the diagnosis of PJI. An increase in the sensitivity from 66.7 to 91% was noted when comparing the results of frozen sections with fixed paraffin embedded histology. Banit et al. [19] studying the results of frozen section histology in 55 knee arthroplasties, 9 of which were infected by microbiological criteria, found that, using the criteria of 10 NPs per HPF, all infected cases were positive on histology; with two false positives being noted. Most other studies on hip and knee arthroplasties have noted high specificity and low/moderate sensitivity for histology to predict positive microbiology in SIF cases [20, 21].

The diagnostic utility of histology is well-illustrated by the findings of our study where 3+ NPs was noted in almost all SIF cases (due to infection with both high- and low-grade organisms); in addition, in all SIF cases where an organism was not cultured, 3+ NPs were seen. Histology was also useful in the diagnosis of AIF, particularly in those cases where an organism was cultured in one sample only. It was noted that in most AIF cases, NPs were absent (84.6%), or present in low numbers (< 1 NP per HPF) (12.8%) with only two cases (2.6%) showing 1–5 NPs per HPF. Thus, the cut-off figure of five NPs per HPF on average adopted by the MSIS correlated well in our study with the final clinical diagnosis of knee SIF and AIF.

Our findings show that all cases which fulfilled the MSIS microbiological criteria for a definite diagnosis of PJI, i.e. growth of a phenotypically indistinguishable pathogen from two or more independent operative specimens, had a final clinical diagnosis of SIF. However, we noted that a significant proportion (20.0%) of knee SIF cases did not meet the threshold for these criteria which is set at this level in order to exclude cases where there is growth of a presumed contaminant organism from a single sample. We found that 23.1% of AIF cases and 8.3% of SIF cases had growth of an organism from one sample. Our findings should be considered with regard to limitations of this retrospective study, notably the fact that the diagnostic criteria for SIF were multiple, non- uniform and to some extent dependent on clinical interpretation [22]. PJI is a condition in which there may be very low numbers of bacteria, some of which are fastidious organisms that are difficult to grow even with prolonged culture regimens and enriched media [2, 3, 15]. Failure to culture organisms microbiologically may be due to a number of factors including low numbers of pathogenic organisms, sampling error and treatment with antibiotics prior to sampling. The sensitivity of microbiological tissue cultures to diagnose PJI in biopsies of periprosthetic tissue has been reported to range from 65 to 94% [22]; several studies have noted that failure to culture an organism from intraoperative specimens does not exclude the diagnosis of PJI [15, 23–25]. It has been shown that in an infected patient, even when four samples are taken, that there is still a 3% chance that all will be culture-negative and a 60% chance that only a single specimen will be culture-positive [15]. Results of microbiology are none the less essential for the identification of causative organisms in PJI and their antibiotic sensitivity.

In conclusion, the results of this study show that the final clinical diagnosis of knee SIF is more often confirmed by MSIS histological than microbiological criteria. It is important to recognize that microbiology results can be negative in cases of knee PJI and that the histological findings of > 5 NPs per HPF is particularly useful in confirming the clinical diagnosis of SIF in this context. In addition, where an organism is cultured from one sample, histology aids in the distinction of knee AIF from SIF, showing few or no NPs in AIF and a significant NP infiltrate in SIF. Correlation of histology and microbiology with other laboratory findings (eg CRP) may improve PJI diagnosis.
